# Characterization, Combustion Behaviour, and Kinetic and Thermodynamic Modelling of Mango Peel as a Potential Biomass Feedstock

**DOI:** 10.3390/polym17131799

**Published:** 2025-06-27

**Authors:** Mohamed Anwar Ismail, Ibrahim Dubdub, Suleiman Mousa, Zaid Abdulhamid Alhulaybi Albin Zaid, Majdi Ameen Alfaiad

**Affiliations:** 1 Mechanical Engineering Department, King Faisal University, Al-Ahsa 31982, Saudi Arabia; maismail@kfu.edu.sa; 2 Chemical Engineering Department, King Faisal University, Al-Ahsa 31982, Saudi Arabia; saamousa@kfu.edu.sa (S.M.); malfaiad@kfu.edu.sa (M.A.A.)

**Keywords:** mango peel, biomass combustion, TGA, kinetics, model-free, model-fitting, thermodynamic parameters, hemicellulose, cellulose, lignin

## Abstract

Mango peel (MP), an abundant agro-industrial residue, was evaluated as a solid biofuel using combined physicochemical characterisation and non-isothermal thermogravimetric kinetics (TGA). Fourier transform infrared (FTIR), X-ray diffraction (XRD), and scanning electron microscopy (SEM) revealed hydroxyl-rich surfaces and porous microstructures. Thermogravimetric combustion, conducted at heating rates of 20–80 K min^−1^, displayed three distinct stages. These stages correspond to dehydration (330–460 K), hemicellulose/cellulose oxidation (420–590 K), and cellulose/lignin oxidation (540–710 K). Kinetic analysis using six model-free methods (Friedman (FR), Flynn–Wall–Ozawa (FWO), Kissinger–Akahira–Sunose (KAS), Starink (STK), Kissinger (K), and Vyazovkin (VY)) yielded activation energies (*E_a_*) of 52–197 kJ mol^−1^, increasing with conversion (mean *E_a_* ≈ 111 kJ mol^−1^). Coats–Redfern (CR) fitting confirmed a three-dimensional diffusion mechanism (D3, R^2^ > 0.99). Thermodynamic analysis revealed that the formation of the activated complex is endothermic, with activation enthalpy (ΔH) values of 45–285 kJ mol^−1^. The process was found to be non-spontaneous under the studied conditions, with Gibbs free energy (ΔG) values ranging from 83 to 182 kJ mol^−1^. With a high heating value (HHV) of 21.9 MJ kg^−1^ and favourable combustion kinetics, MP is a promising supplementary fuel for industrial biomass boilers. However, its high potassium oxide (K_2_O) content requires dedicated ash management strategies to mitigate slagging risks, a key consideration for its practical, large-scale application.

## 1. Introduction

The global energy sector is experiencing a critical shift as fossil fuel depletion and environmental concerns, including greenhouse gas emissions and air pollution, drive the transition to renewable energy sources [1]. Biomass, a widely available and carbon-neutral resource, stands out as a promising solution to boost energy security and promote sustainability [2]. Among biomass resources, agro-industrial wastes are particularly attractive, offering a dual benefit of clean energy generation and waste minimization by repurposing materials that would otherwise pose environmental risks [3]. Lignocellulosic biomass, composed of cellulose, hemicellulose, and lignin, is especially suitable for thermochemical conversion processes such as combustion, pyrolysis, and gasification [4,5].

MP, an abundant by-product of mango (Mangifera indica) processing, represents a significant agro-industrial waste stream in tropical and subtropical regions. In Egypt, for example, over 1.5 million tons of mangoes were harvested in 2020, producing considerable peel waste [6]. With a lignocellulosic composition of 12.02 wt% hemicellulose, 17.02 wt% cellulose, and 10.0 wt% lignin, along with a high HHV, MP shows strong potential as a biofuel feedstock [7]. However, its ash content and elevated alkali metal levels within that ash, such as potassium and calcium, pose challenges like slagging and fouling during combustion, necessitating thorough characterization and combustion studies to evaluate its practical feasibility [8].

Numerous studies have explored the thermal decomposition and kinetic behaviour of lignocellulosic biomass using TGA, focusing on various feedstocks and thermochemical processes. Poletto et al. used TGA to evaluate the thermal stability of wood sawdust from different species, highlighting the influence of lignocellulosic content on decomposition behaviour and estimating activation energies between 146 and 165 kJ mol^−1^ via FWO and Kissinger methods [9]. Similarly, Nath et al. investigated wheat straw pellet combustion using model-free and model-fitting approaches, revealing a multi-stage decomposition process and average activation energies exceeding 160 kJ mol^−1^ [10]. Barzegar et al. studied the TGA and kinetics of torrefied pine wood under air and oxy-fuel conditions, reporting *E_a_* values ranging from 100 to 250 kJ mol^−1^ [4]. Mohammed et al. characterized Napier grass as a feedstock, highlighting its high volatile matter and favourable combustion properties [3]. Liu et al. analysed the pyrolysis and combustion of corn straw, poplar wood chips, and rice husks, noting *E_a_* variations due to compositional differences [8].

Specific to mango-derived biomass, Okoroigwe and Okoroigwe et al. investigated the combustion of mango wood and African bush mango shell using TGA at a heating rate of 30 K min^−1^, identifying two-stage decomposition (volatile oxidation and char combustion) with *E_a_* values of 99–404 kJ mol^−1^ using the Coats–Redfern (CR) method [11,12]. Arenas et al. studied the pyrolysis kinetics of MP using five model-free methods (Friedman, KAS, FWO, Vyazovkin, Starink) at heating rates of 5–20 K min^−1^, reporting high *E_a_* values (150–530 kJ mol^−1^) [5]. El-Sayed and Mostafa explored the pyrolysis kinetics of mango leaves, applying isoconversional and parallel reaction models [13]. Souza et al. conducted a thermal analysis of MP under nitrogen, observing multiple decomposition stages due to its complex lignocellulosic structure [7]. Tahir et al. performed TGA on MP pyrolysis at 10–30 K min^−1^, identifying three decomposition stages: dehydration (297–458 K, 4% weight loss), hemicellulose and cellulose degradation (435–589 K, 31% weight loss), and lignin and char degradation (563–711 K, 30% weight loss), while calculating *E_a_* values of 88–304 kJ mol^−1^ using KAS, Starink, and FWO methods [14]. Similarly, Yousef et al. analysed mango seed shells under N_2_ and CO_2_ atmospheres at 5–30 K min^−1^, reporting three stages—dehydration (298–463 K, 9% weight loss), hemicellulose and cellulose degradation (471–645 K, 58% weight loss), and lignin and char degradation (>711 K, 24% weight loss)—with *E_a_* values of 50–550 kJ mol^−1^ using multiple methods (Friedman, KAS, FWO, DAEM, Vyazovkin) [15]. Alves et al. studied mango seed pyrolysis at 5–30 K min^−1^, identifying three decomposition stages with *E_a_* values varying across methods [16].

Most mango biomass work to date has been limited to slow laboratory heating rates (≤30 K min^−1^) and has focused mainly on inert atmosphere pyrolysis [5,14,15,16]. However, oxidative experiments at higher heating rates are far less common. These conditions, where devolatilisation is rapid and reaction stages overlap, are more representative of industrial processes, yet limited combustion data exist for MP under such scenarios. Because reaction rates and apparent activation energies depend strongly on heating rate, such gaps leave uncertainty in designing small-scale burners or co-firing schemes that might employ this residue. In addition, although several authors have reported FTIR, XRD, SEM, or XRF data for MP or seed fractions [7,16], few studies place these structural insights alongside elevated-rate combustion kinetics and supporting thermodynamic calculations.

The study aims to fill this knowledge gap by providing a comprehensive analysis of MP combustion under industrially relevant heating rates (20, 40, 60, and 80 K min^−1^). The primary objectives are (1) to perform a thorough physicochemical and morphological characterization of MP (proximate, ultimate, fibre, FTIR, XRD, SEM, XRF); (2) to determine its combustion behaviour and kinetics using six isoconversional (model-free) and Coats–Redfern (model-fitting) methods; and (3) to calculate key thermodynamic parameters to assess reaction feasibility. By integrating these results, this work supplies the first comprehensive dataset for MP combustion at elevated rates, providing essential inputs for designing and optimizing biofuel applications for this abundant agro-industrial waste. This research is part of a broader investigation into agricultural wastes, with future work planned on pyrolysis and co-pyrolysis to further explore their valorisation potential.

## 2. Materials and Methods

### 2.1. MP Collection and Sample Preparation

Mangoes (Alphonso variety) used in this study were sourced from the Ismailia governorate in Egypt, a region known for its favourable soil and climate for mango cultivation. Whole mangoes were washed with distilled water to remove surface contaminants, including dirt and microbial impurities, and then manually peeled to isolate the peel for analysis. The peel was retained as the only material for analysis in this study due to its potential as an agro-waste for biofuel applications. The peel was dried at 310 K for 24 h in a hot air oven (Memmert UN110, Büchenbach, Germany), ground using a laboratory mill (IKA MF 10 Basic, Staufen, Germany), and sieved with an ELE Sieve Shaker (code: 80-0200/0) (ELE International, Milton Keynes, UK) to achieve a uniform particle size of 0.34 ± 0.05 mm. This particle size was selected to ensure consistent heat transfer and reproducible thermogravimetric results, critical for accurate kinetic and combustion studies. The prepared samples were stored in airtight containers at 298 K under dry conditions to prevent moisture reabsorption. The characteristics of the dried, milled MP are summarised in Table 1. All measurements were conducted in triplicate.

### 2.2. Physico-Chemical Characterization

#### 2.2.1. Proximate Analysis

Proximate analysis was conducted to determine the weight percentages of moisture, volatile matter, ash, and fixed carbon contents using a thermogravimetric analyser (TGA-2 Star System, Mettler Toledo, Greifensee, Switzerland). Approximately 10 mg of MP was heated under a controlled atmosphere: first at 383 K for 30 min under nitrogen to determine moisture content, then at 1173 K for 7 min under nitrogen to measure volatile matter, and finally at 823 K for 15 min under O_2_ to quantify ash content. Fixed carbon was calculated by difference:

(1) Fixed Carbon (%)=100−(Moisture+Volatile Matter+Ash)

#### 2.2.2. Ultimate Analysis

Ultimate analysis was carried out using a CHNS elemental analyser (Vario EL III, Elementar, Langenselbold, Germany) to quantify carbon, hydrogen, nitrogen, and sulphur contents on a dry basis. Approximately 2 mg of MP was combusted at 1273 K under an oxygen atmosphere, with combustion gases analysed via thermal conductivity detection. Oxygen content was calculated by difference using dry basis values:

(2) Oxygen (%)=100−(C+H+N+S+Ash)

#### 2.2.3. Fibre Analysis

Fibre composition (cellulose, hemicellulose, lignin) was determined using the Van Soest method with an ANKOM 2000 Automated Fiber Analyzer (ANKOM Technology, Macedon, NY, USA). Approximately 0.5 g of MP was treated with a neutral detergent solution to determine neutral detergent fibre (NDF), followed by acid detergent solution for acid detergent fibre (ADF) and 72% sulphuric acid for acid detergent lignin (ADL, lignin). Hemicellulose and cellulose contents were as follows:

(3) Hemicellulose = NDF − ADFCellulose=ADF−ADL

#### 2.2.4. Higher Heating Value (HHV)

The HHV was measured using a Plain Oxygen Bomb Calorimeter (Model 1341EE, Parr Instrument Company, Moline, IL, USA). A 0.5 g pelletized MP sample was combusted under 3 MPa oxygen pressure, with the temperature rise calibrated against benzoic acid. Measurements were conducted in triplicate.

### 2.3. Structural and Elemental Characterization

#### 2.3.1. Fourier Transform Infrared (FTIR) Spectroscopy

FTIR spectroscopy was performed using an FT/IR-4000 spectrometer (JASCO, Tokyo, Japan) to identify functional groups. Approximately 2 mg of MP was mixed with 200 mg of KBr, pressed into a pellet, and scanned over 4000–399.19 cm^−1^ at a resolution of 4 cm^−1^ with 32 scans. Spectra were corrected for H_2_O and CO_2_ interference using the instrument’s built-in software.

#### 2.3.2. X-Ray Diffraction (XRD) Analysis

XRD analysis was conducted using an X-ray diffractometer (Rigaku Ultima IV, Akishima, Japan) with Cu Kα radiation (λ = 1.54 Å) at 40 kV and 30 mA. MP samples were scanned from 2θ = 10° to 90° at a rate of 1° min^−1^ with a step size of 0.02°. The crystallinity index (CrI) was calculated using the Segal method:

(4) $$\mathrm{CrI}(\%)=\left(\frac{I_{002}-I_{am}}{I_{002}}\right)\times 100$$

where $I_{002}$ is the intensity of the cellulose-I peak at $2\theta \approx 22^{\circ }$, and $I_{\mathrm{am}}$ is the intensity of the amorphous halo at 2θ ≈ 18°. Mineral interferences were identified and corrected following standard procedures.

#### 2.3.3. X-Ray Fluorescence (XRF) Analysis

Ash composition was analysed using an XRF spectrometer (Lab-X3500, Oxford Instruments, Abingdon, UK). MP ash was prepared by combusting 5 g of sample at 973 K according to ASTM D3174-11 [17], pressed into a pellet with boric acid binder, and analysed under vacuum. Major elements (e.g., K, Ca, Si, S) were quantified using a standard less calibration method, with measurements conducted in triplicate.

#### 2.3.4. Scanning Electron Microscopy (SEM)

Surface morphology and porosity were examined using a JEOL JSM-6390LV SEM (JEOL, Akishima, Japan) at an accelerating voltage of 15 kV. MP samples were mounted on aluminium stubs, sputter-coated with a 10 nm gold layer (Quorum Q150R ES, Quorum Technologies, Laughton, UK), and imaged at magnifications corresponding to 100 µm and 10 µm scales.

### 2.4. Thermogravimetric Analysis (TGA)

Combustion behaviour was studied on a NETZSCH STA 449 F3 Jupiter simultaneous TG–DSC analyser (NETZSCH-Gerätebau, Selb, Germany). Approximately 10 mg of MP was placed in an Al_2_O_3_ crucible and heated from 303 K to 973 K at heating rates of 20, 40, 60, and 80 K min^−1^ under a synthetic air atmosphere (80% N_2_, 20% O_2_) at a flow rate of 60 mL min^−1^. Mass loss (TG) and derivative mass loss (DTG) profiles were recorded. Experiments were conducted in triplicate.

### 2.5. Kinetic and Thermodynamic Modelling

#### 2.5.1. Kinetic Analysis

The combustion kinetics of MP were evaluated by the same protocol we applied previously to orange peel and PVA polymer [2,18]. TG data recorded at 20, 40, 60, and 80 K min^−1^ were converted to conversion (α) and analysed between α = 0.10 and α = 0.60. Six isoconversional (model-free) methods—FR, FWO, KAS, STK, K, and VY)—were implemented to obtain apparent activation energies *E_a_*(*α*). Details of the model-free and model-fitting methods used for the kinetic analysis of MP combustion are provided in Table S1 in the Supplementary Materials. The CR model fitting approach screened sixteen solid-state reaction models (e.g., reaction order, diffusion, nucleation, and geometrical contraction models), as listed in Table S2 (Supplementary Materials).

#### 2.5.2. Thermodynamic Parameters

The thermodynamic parameters—enthalpy (ΔH), Gibbs free energy (ΔG), and entropy (ΔS)—of MP combustion were calculated following the equations and methods similar to those outlined in previous works [2,18] and are shown below:

(5) $$∆H=E_{a}-R\;T_{p}$$

(6) $$∆G=E_{a}+R\;T_{p}ln\left(\frac{k_{B}\;T_{p}}{h\;A_{0}}\right)$$

(7) $$∆S=\frac{∆H-∆G}{T_{p}}$$

where $T_{p}$ represents the maximum temperature (K), $k_{B}$ is the Boltzmann constant (1.381 × 10^−23^ J K^−1^), h is the Planck constant (6.626 × 10^−34^ J s), and $A_{o}$ is the pre-exponential factor, (min^−1^). These parameters were computed across conversions to evaluate the feasibility of MP combustion.

## 3. Results and Discussion

### 3.1. Fundamental Properties of MP

#### Proximate, Ultimate, and Fibre Analysis: Implications for Combustion Efficiency

The proximate analysis of MP (Table 1) showed a moisture content of 6.0 ± 0.02 wt% on an air-dry basis. On a dry basis, MP exhibited a high volatile matter content (71.91 ± 0.11 wt%), indicative of favourable ignition characteristics and efficient combustion. The ash content was determined to be 7.55 ± 0.03 wt% (dry basis), which is moderate for biomass and suggests that ash management strategies may be necessary for continuous combustion operations. The fixed carbon content was 20.53 ± 0.10 wt% (dry basis), contributing to sustained energy release during char combustion. These proximate values are generally in line with those of other agricultural residues, although the ash content is higher than the 3.23% reported by Tahir et al. [14] for their MP sample, highlighting natural compositional variability.

The ultimate analysis indicated a carbon content of 44.15 ± 0.16 wt% and hydrogen at 6.28 ± 0.03 wt%. The low sulphur content (0.33 ± 0.04 wt%) is advantageous, implying minimal SO_x_ emissions during combustion. The nitrogen content was 2.45 ± 0.10 wt%. When compared to orange peel (OP), which has a reported volatile matter of 69.5 wt%, the MP in this study shows a similarly high volatile matter content (71.91 wt%, dry basis), suggesting comparable devolatilization behaviour [2]. The fibre composition of MP was found to be 12.02 ± 0.04 wt% hemicellulose, 17.02 ± 0.12 wt% cellulose, and 10.00 ± 0.05 wt% lignin. These values for the main lignocellulosic components sum to 39.04 wt% of the dry biomass. These fractions are somewhat lower than those reported by Tahir et al. for MP (hemicellulose 23.9%, cellulose 31.5%, lignin 38.8%). Such variations can arise from differences in mango variety, ripeness, geographical origin, and the analytical methodologies employed for fibre determination, particularly how components like pectins (abundant in fruit peels) are fractionated or excluded.

The higher heating value (HHV) of MP was measured at 21.9 ± 0.05 MJ kg^−1^. This energy content is competitive with other common biomass fuels, such as rice husk (15–20 MJ kg^−1^) [8], and is substantially higher than the 16.13 MJ/kg reported by Tahir et al. [14] for their MP sample. The higher carbon content and lower ash content relative to some biomass contribute to this favourable HHV, highlighting MP’s potential as an energy source. However, the 7.55 wt% ash content necessitates careful consideration in combustion system design to mitigate potential operational issues.

### 3.2. Structural, Elemental, and Morphological Insights

#### 3.2.1. FTIR Spectroscopy: Functional Group Identification

**Figure 1:**
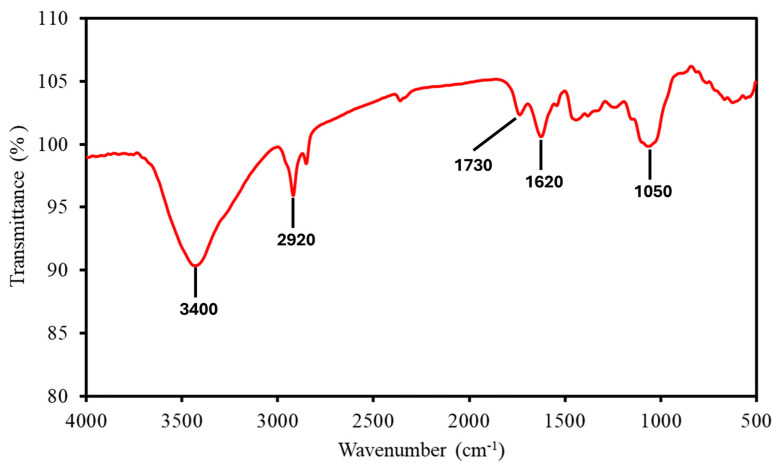
FTIR spectrum of MP, showing key absorption peaks labelled.

FTIR spectroscopy (Figure 1) shows the key functional groups in the MP sample reflecting its lignocellulosic composition. The broad absorption peak at 3400 cm^−1^ corresponded to O-H stretching vibrations, indicative of hydroxyl groups in cellulose and hemicellulose, which contribute to MP’s hygroscopic nature and influence dehydration during early combustion stages. The peak at 2920 cm^−1^ represented C-H stretching of aliphatic groups, associated with the organic backbone of lignocellulosic components, facilitating volatile release during thermal decomposition. The C=O stretching at 1730 cm^−1^ and C=C stretching at 1620 cm^−1^ confirmed the presence of lignin, with the C=O indicating carbonyl groups in lignin’s structure and C=C reflecting its aromatic framework, both contributing to thermal stability during combustion. The peak at 1050 cm^−1^ corresponded to C-O stretching in cellulose and hemicellulose, signifying ether and alcohol groups that decompose readily during oxidative processes. These functional groups collectively influenced MP’s thermal stability and combustion reactivity, consistent with findings for similar biomass [15].

#### 3.2.2. XRD Analysis: Crystallinity and Mineral Phases

**Figure 2:**
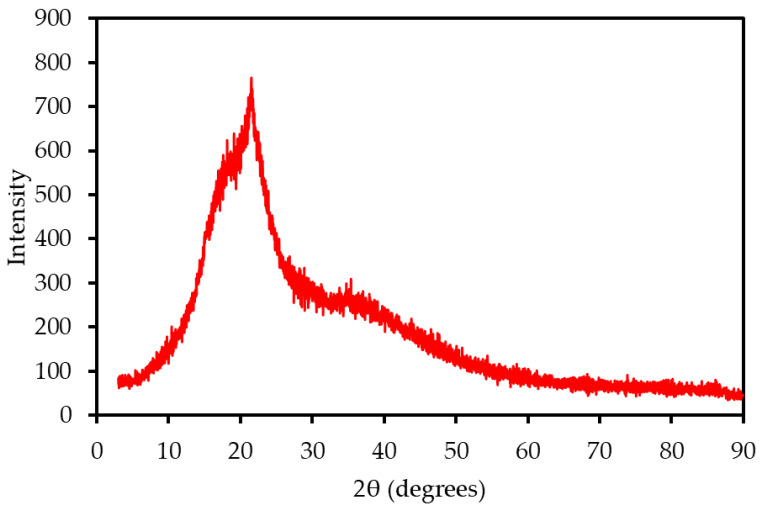
XRD pattern of MP, showing cellulose I peaks and mineral phases.

The XRD pattern of MP (Figure 2) exhibited characteristic peaks of cellulose I at 2θ ≈ 16° and 22°, with an amorphous halo at 2θ ≈ 18°, reflecting the lignocellulosic structure of the biomass. The crystallinity index (CrI), calculated using the Segal method, was approximately 45%, indicating a moderate crystalline cellulose content, lower than that of wood (CrI ~60%; Tahir et al., 2021 [14]). This moderate crystallinity suggests a balanced thermal decomposition profile, as crystalline cellulose decomposes more slowly than amorphous components, potentially stabilizing combustion. A distinct peak at 2θ ≈ 29° was identified as calcite (CaCO_3_), consistent with the high calcium content (16.66 wt%) observed in XRF analysis. The presence of calcite indicates potential ash-related challenges, such as slagging, which may require mitigation strategies in combustion systems.

#### 3.2.3. X-Ray Fluorescence (XRF) of Ash

The ash composition of MP, determined by XRF spectroscopy, is detailed in Table 2, with results presented on an oxide basis alongside corresponding elemental percentages. Key components include potassium oxide (K_2_O: 40.9 wt%), calcium oxide (CaO: 23.3 wt%), and silica (SiO_2_: 11.06 wt%). These elemental proportions (K, Ca, Si as wt% of ash) are consistent with the data presented in Table 1 under “Metallic Elements (wt% of ash)”. The high concentration of K_2_O in the ash signals a potential risk for ash deposition, slagging, and corrosion in combustion systems. However, the overall moderate ash content of the MP may help to lessen these risks. The sulphur content in the ash, derived from SO_3_, is significantly higher than the total sulphur content of the original MP biomass (0.33 wt% on a dry basis, Table 1). This suggests that the sulphur quantified by XRF in the ash (prepared at 973 K) likely represents a concentration of less volatile sulphur compounds or could be influenced by reactions during ashing or analytical factors, rather than indicating that a large fraction of the biomass’s original sulphur is retained in the ash as SO_3_. Given the very low total sulphur in the MP, SO_2_ emissions during combustion are expected to be minimal, though appropriate emission control measures should always be considered in practical applications.

#### 3.2.4. SEM Analysis: Surface Morphology and Porosity

**Figure 3:**
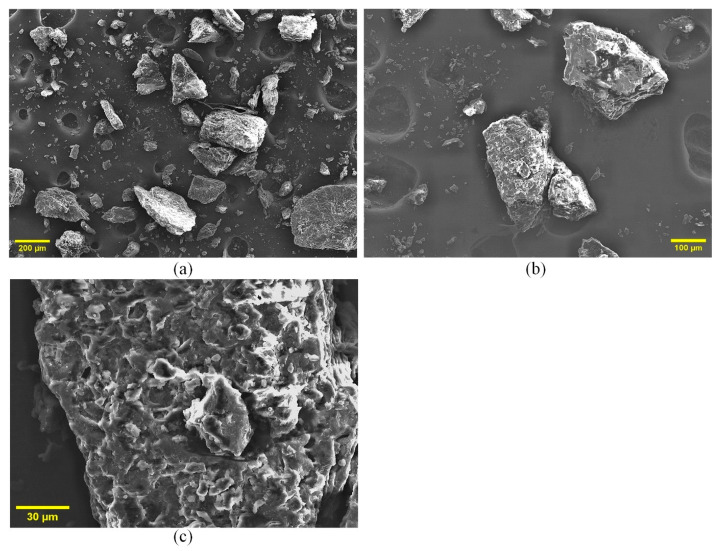
SEM images of MP at (**a**) 200 µm, (**b**) 100 µm, and (**c**) 30 µm scales, showing surface morphology and porosity.

SEM analysis revealed the surface morphology of the MP powder (Figure 3). The particles are irregular and fragmented, a result of mechanical grinding that increases the surface area for reaction (Figure 3a). At higher magnifications (Figure 3b,c), the surface appears fibrous and highly porous, with a network of cracks and interconnected pores. This microstructure is critical for combustion efficiency, as it facilitates oxygen diffusion into the particle and the escape of volatile gases. The inherent porosity directly supports the three-dimensional diffusion (D3) mechanism identified as the rate-limiting step in our kinetic analysis, confirming that mass transport through this porous matrix governs the overall reaction rate.

#### 3.2.5. Combustion Characteristics via TGATG-DTG Profiles: Decomposition Stages and Influence of Heating Rate

**Figure 4:**
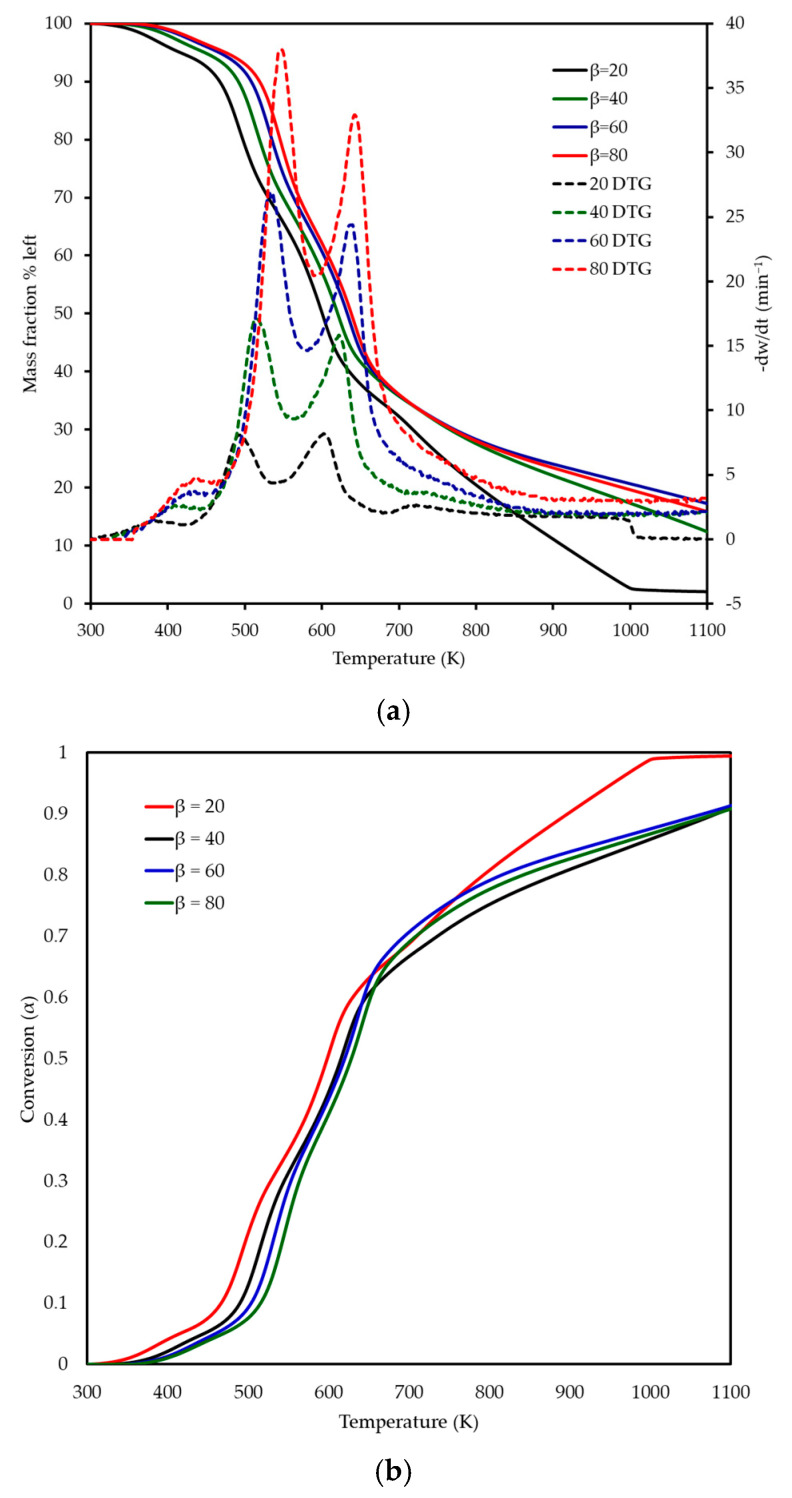
(**a**) Thermogravimetric (TG) and (**b**) derivative thermogravimetric (DTG) curves, along with conversion profiles, for MP combustion at four different heating rates (20, 40, 60, and 80 K min^−1^).

The thermogravimetric and derivative thermogravimetric profiles of MP combustion at heating rates of 20, 40, 60, and 80 K min^−1^ are shown in Figure 4a,b, illustrating the mass fraction remaining and derivative mass. As the heating rate increased, a shift in the TG and DTG curves to higher temperatures was observed, attributed to reduced heat transfer efficiency at higher rates, leading to delayed decomposition. Three distinct decomposition stages were identified between 300 and 800 K, as detailed in Table 3 and reflected in Figure 4a. “T range” in Table 3 refers to the initial and final temperature of each reaction. The first stage, corresponding to dehydration, occurred over temperature ranges of 340–420 K, 344–442 K, 348–460 K, and 352–464 K at heating rates of 20, 40, 60, and 80 K min^−1^, respectively, with a mass loss of 3–6%, which appears as a subtle initial decline in the TG curves and a small DTG peak (Figure 4). The second stage involved the degradation of hemicellulose and a significant portion of cellulose, occurring over 420–538 K, 442–560 K, 460–580 K, and 464–590 K, with a mass loss of 22–35% observed as a prominent DTG peak shifting from 495 K at 20 K min^−1^ to 550 K at 80 K min^−1^, reflecting the heating rate effect. The third stage encompassed the decomposition of remaining cellulose and lignin, spanning 538–680 K, 560–700 K, 580–703 K, and 590–705 K, with a mass loss of 33–47% identified by a broader DTG peak at 603–642 K, extending toward 700 K at higher rates and indicating the thermal stability of lignin. These stages aligned with the inflection points and peak maxima in the TG and DTG curves of Figure 4, confirming the sequential decomposition of MP’s lignocellulosic components. The final residual mass varied slightly with the heating rate, from ~7 wt% at 20 K min^−1^ to ~8 wt% at 80 K min^−1^. This variation is attributed to heat and mass transfer limitations. At lower heating rates, the longer residence time at high temperatures allows for more complete oxidation of the fixed carbon. On the other hand, at higher heating rates, steep thermal gradients can lead to incomplete char burnout by the time the experiment terminates at 973 K. This phenomenon, where final residue depends on heating rate, is consistent with reports for other lignocellulosic fuels [10]. The higher heating rates (up to 80 K min^−1^) revealed intensified thermal behaviour, not fully captured by prior studies at lower rates (5–20 K min^−1^) [14,15], emphasising the importance of investigating combustion under conditions closer to industrial thermal processing environments. No recent publications on MP combustion by TGA were found for direct comparison, highlighting the novelty of these findings.

### 3.3. Kinetic Modelling of MP Combustion

#### 3.3.1. Activation Energies from Model-Free Methods

**Figure 5:**
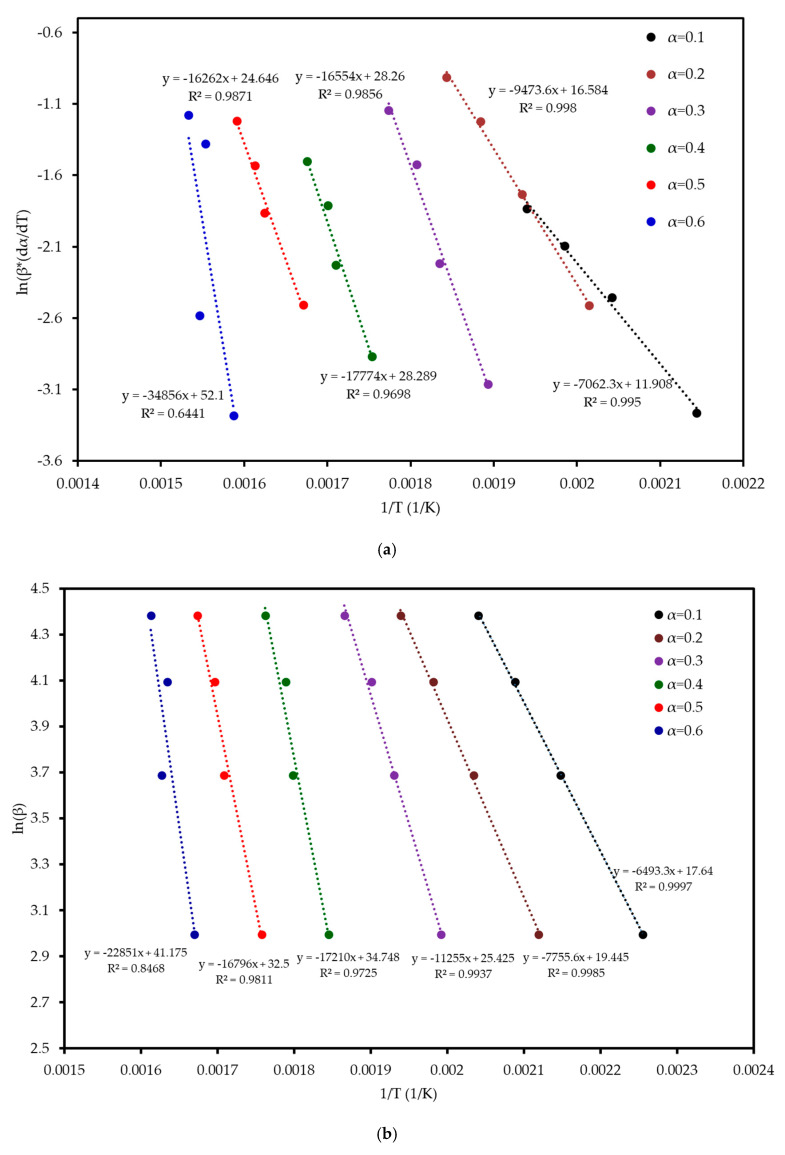

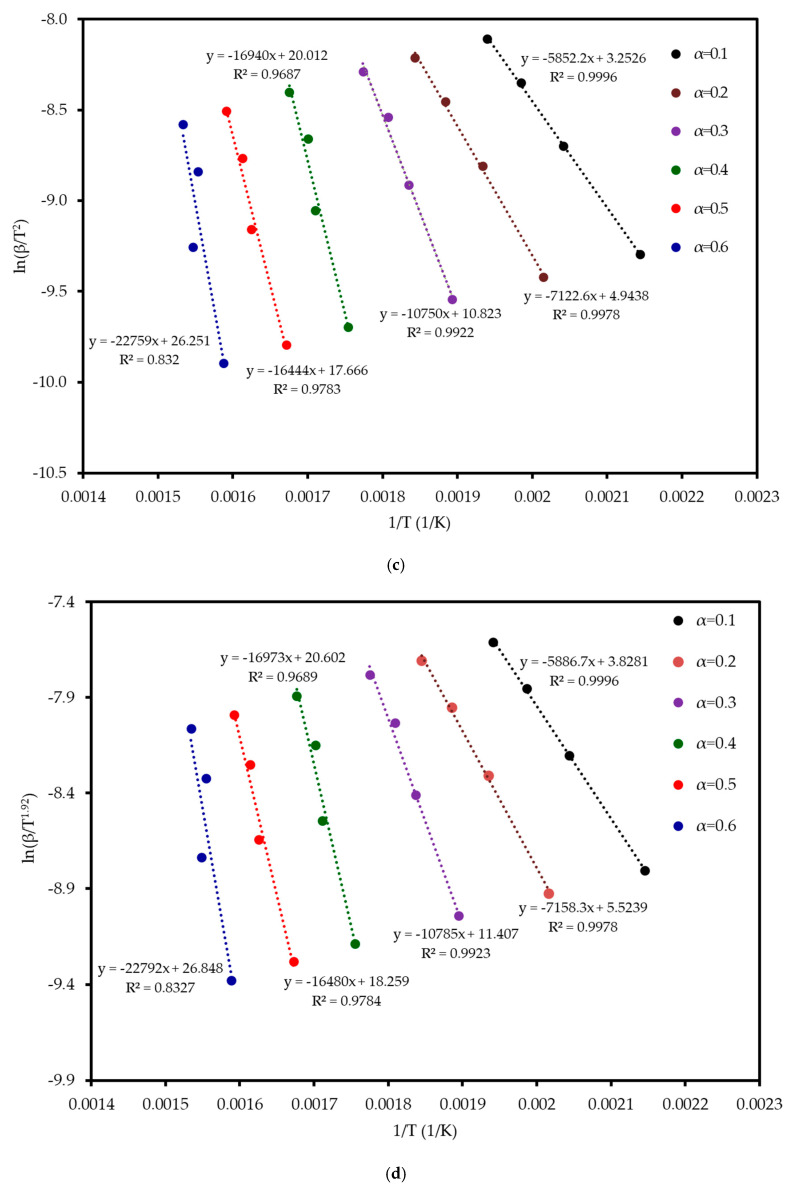

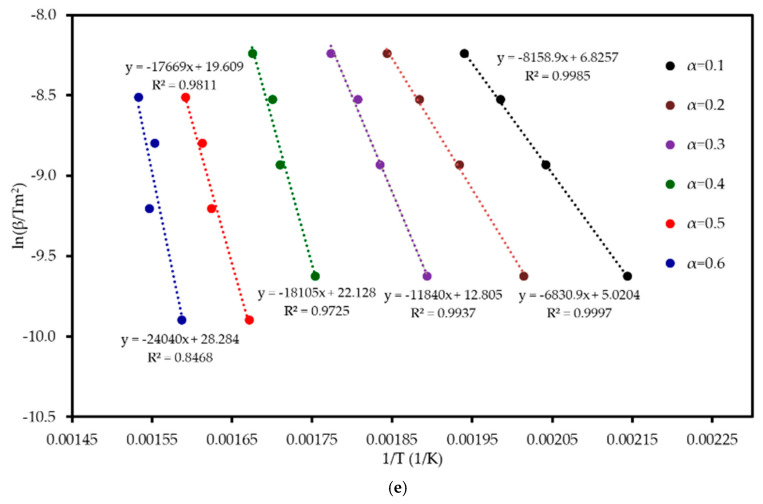
Regression analysis of MP combustion using five model-free methods: (**a**) FR, (**b**) FWO, (**c**) KAS, (**d**) STK, and (**e**) K.

**Figure 6:**
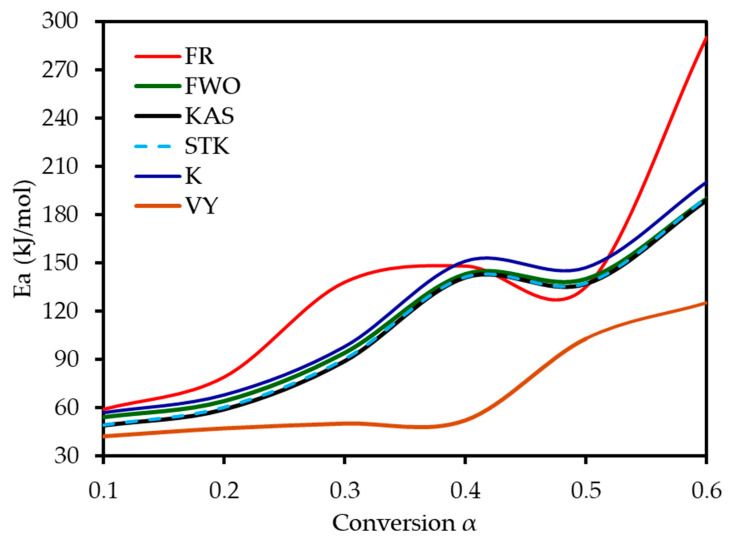
Activation energy values of MP combustion determined using six model-free methods.

Six model-free methods—FR, FWO, KAS, STK, K, and VY—were applied to estimate the activation energy (*E_a_*) of MP combustion over a conversion range of 0.1–0.6. This range was selected because beyond α ≈ 0.6, the kinetic analysis becomes unreliable. In this final stage, the DTG signal flattens, and baseline drift can become significant relative to the small amount of remaining mass. Furthermore, overlapping reactions of char oxidation and the decomposition of inorganic minerals can distort the single-step reaction assumption required by the model-free algorithms. Following ICTAC kinetic analysis guidelines, this region was truncated to avoid introducing artificial oscillations into the calculated activation energy values. These methods, endorsed by the ICTAC Kinetics Committee [19], rely on data from multiple heating rates and are independent of predefined reaction mechanisms. Linear regression plots (Figure 5) of ln(β dα/dT), ln(β), ln(β/T^2^), ln(β/T^1^·^92^), and ln(β/T_m^2^) versus 1/T for the respective methods showed a leftward shift with increasing conversion, indicating a progressive increase in *E_a_*. The *E_a_* increased from an average of 52 kJ mol^−1^ at α = 0.1 to 197 kJ mol^−1^ at α = 0.6, reflecting the transition from dehydration (low *E_a_*) to hemicellulose/cellulose degradation (moderate *E_a_*) and lignin degradation (high *E_a_*). This trend aligns with the complex chemical structure of MP, where hemicellulose and cellulose combust at lower temperatures with lower *E_a_*, while lignin’s cross-linked aromatic structure requires higher energy. From Table 4 and Figure 6, it can be seen that the FR method yielded the highest *E_a_* range (59–290 kJ mol^−1^, average 142 kJ mol^−1^), while FWO, KAS, STK, and K methods showed similar values (54–200 kJ mol^−1^, average 116 kJ mol^−1^), and VY exhibited a narrower range (55–210 kJ mol^−1^, average 114 kJ mol^−1^). The FR method, being a differential isoconversional method, is highly sensitive to fluctuations in the rate of mass loss, which can lead to higher and more variable *E_a_* values compared to the more stable integral methods (FWO, KAS, STK). Compared to MP pyrolysis studies (Tahir et al., 2021) [14], which reported *E_a_* values of 88–304 kJ mol^−1^ using KAS, STK, and FWO at lower heating rates (10–30 K min^−1^), the present study’s higher heating rates (20–80 K min^−1^) and additional methods provide a more comprehensive kinetic profile, enhancing applicability to industrial combustion processes.

#### 3.3.2. Reaction Mechanism and Kinetic Parameters from Coats–Redfern Method

The Coats–Redfern method was employed to determine the reaction mechanism and kinetic parameters of MP combustion, using a single heating rate per experiment (Coats and Redfern, 1965) [20]. Linear regression of $ln\left[g(\alpha )/T^{2}\right]$ versus 1/T was performed for 15 solid-state reaction models (Table S2 in Supplementary Materials) across four heating rates, corresponding to the three reaction stages identified in Table 3. The resulting $E_{a}$, pre-exponential factor $\left(lnA_{0}\right)$, and $R^{2}$ values are summarized in Table S3 (in the Supplementary). Diffusion models, particularly D3, consistently achieved $R^{2}>0.96$, confirming the reliability of the model fitting approach for these mechanisms, while nucleation models (e.g., A4, P4) often showed lower $R^{2}$ values (<0.96) [21]. The three-dimensional diffusion model (D3) consistently provided the highest $R^{2}$ values (>0.9936, highlighted in bold in Table S3) indicating that diffusion within an evolving char matrix is rate-limiting during mid to late conversion. While D1, D2, and D4 also achieved R^2^ values above 0.996, D3 stands out with a mean R^2^ of approximately 0.9980, indicating a statistically superior fit. Its three-dimensional geometry aligns with the porous char structure seen in SEM, and its *E_a_* range (90–122 kJ mol^−1^) matches the model-free average (111 kJ mol^−1^) within ±10%, unlike the >15% deviations of other models. Thus, D3 was chosen for thermodynamic analysis.

The D3 model was selected for calculating thermodynamic parameters (ΔH, ΔG, and ΔS) using its integral function $g(\alpha )={\left[1-(1-\alpha {)}^{1/3}\right]}^{2}$ and differential function $f(\alpha )=\frac{3}{2}{\left[1-(1-\alpha {)}^{1/3}\right]}^{-1}$, as presented in Table S3. For example, in the second reaction stage (hemicellulose and cellulose degradation), $E_{a}$ values for the D3 model ranged from $90\;kJ{\;mol}^{-1}\left(20\;K{\;min}^{-1}\right)$ to $122\;kJ{\;mol}^{-1}\left(80\;K{\;min}^{-1}\right)$, aligning with the model-free $E_{a}$ averages for mid-range conversions (Table 3). This diffusion-controlled mechanism reflects the transport of reactants and products through MP’s porous matrix, as observed in SEM analysis, and is consistent with combustion studies of lignocellulosic biomass (Tariq et al., 2022) [22].

In contrast, Okoroigwe et al. [12] applied the CR method to analyse the combustion of mango wood and shell at a single heating rate ($30\;K{\;min}^{-1}$), identifying two reaction stages: volatile decomposition (473–648 K for mango wood, 473–623 K for mango shell) and char combustion (648–773 K for mango wood, 623–748 K for mango shell). They tested reaction order models (n=0,1,2,3), selecting n=1 for mango wood ($E_{a}=99–164\;kJ$ ${mol}^{-1}$) and n=2 for mango shell ($E_{a}=136–404\;kJ{\;mol}^{-1}$) based on the highest $R^{2}$. The broader $E_{a}$ range compared to the current study’s D3 model ($E_{a}=64–122\;kJ{\;mol}^{-1}$) likely results from differences in sample composition (wood/shell vs. peel), the use of a single heating rate, and their limited model selection (four reaction order models vs. 15 models in this study).

### 3.4. Thermodynamic Assessment of Combustion

Thermodynamic parameters—enthalpy (ΔH), Gibbs free energy (ΔG) and entropy (ΔS)—were calculated from the Arrhenius parameters obtained with the five isoconversional methods (FR, FWO, KAS, STK, K). Calculations employed the D3 diffusion model functions f(α) and g(α); the results are compiled in Table 5. In combustion studies, ΔH reveals whether a step is endothermic or exothermic, ΔS reflects the change in system disorder, and ΔG indicates the spontaneity of the overall reaction [23]. All ΔH (activation enthalpy) values are positive, confirming that formation of the activated complex is endothermic, while the overall combustion of MP remains exothermic [24,25]. The small $E_{a}-\Delta H$ difference ($3.37–5.20\;kJ{\;mol}^{-1}$) implies only a modest additional energy requirement to form the transition state, facilitating reaction progress once ignition occurs [26]. ΔG is likewise positive ($82.59–182.06\;kJ{\;mol}^{-1}$); above $130\;kJ{\;mol}^{-1}$ at α≥0.4, it signifies a substantial barrier that demands thermal support during the later char-oxidation stage (Kalidasan et al. 2023) [27]. ΔS changes from negative to positive with increasing conversion. Negative values at low conversions (α = 0.1–0.3) suggest that the activated complex is more ordered than the initial reactants, which is characteristic of the initial devolatilisation and formation of a structured char. In contrast, the positive ΔS value at higher conversion (α = 0.6) indicates increased disorder, reflecting the breakdown of the complex char matrix into gaseous products during the final oxidation stage. This behaviour is typical for the combustion of complex lignocellulosic fuels [24]. These observations underline how kinetic and thermodynamic factors interact in MP combustion, with an endothermic devolatilization phase preceding diffusion-controlled char oxidation.

### 3.5. Integrated Analysis and Implications for Biofuel Applications

MP demonstrates significant potential as a solid biofuel, characterized by a competitive HHV, a high volatile matter content, and a porous microstructure revealed by SEM analysis, all of which contribute to enhanced combustion reactivity. Structural characterization via FTIR and XRD confirmed its lignocellulosic nature, with the primary fibre components being hemicellulose, cellulose, and lignin. The biomass exhibited moderate crystallinity, and the presence of key functional groups influences its thermal stability and decomposition pathways. Thermogravimetric analysis identified three principal decomposition stages during combustion, with apparent activation energies (Eₐ) ranging from 52–197 kJ mol^−1^, indicating that MP combustion is kinetically feasible under typical industrial thermal conditions. The Coats–Redfern method identified a three-dimensional diffusion-controlled mechanism (D3) as best describing the dominant reaction pathway, which aligns with the observed porous matrix of MP that facilitates the transport of reactants and products during conversion. While the energy attributes are favourable, the ash analysis revealed an elevated potassium oxide content (K_2_O representing 34.1 wt% of the 7.55 wt% dry basis ash), which poses a risk of slagging and fouling in combustion equipment. Equally, the substantial calcium oxide (CaO) fraction in the ash may partially mitigate alkali volatilization during combustion. In summary, MP is a high-potential biofuel due to its energy content and reactivity. For industrial applications, its primary limitation is the high potassium content in its ash, which presents a significant slagging and fouling risk. Therefore, practical implementation will likely require co-firing with fuels low in alkali metals or the use of boilers specifically designed for challenging biomass. Future work should focus on pilot-scale combustion trials to validate these kinetic findings, test ash mitigation strategies (e.g., using additives), and perform detailed emissions characterization to ensure environmental compliance.

## 4. Conclusions

This study successfully characterized the combustion of MP biomass, establishing it as a viable but challenging renewable fuel. The investigation at elevated heating rates (20–80 K min^−1^) provided critical insights into its thermal decomposition, which occurs in three main stages. The kinetic analysis confirms that the process is governed by a three-dimensional diffusion mechanism (D3), with activation energies comparable to other agricultural residues. From a broader perspective, these findings highlight that MP combustion offers a sustainable pathway for valorising a significant agro-industrial waste stream, contributing to a circular economy and reducing landfill burden. While its high heating value is attractive, the key challenge for industrial adoption is managing the high potassium content in its ash to prevent operational issues like slagging. Its negligible sulphur content is a significant environmental advantage, promising minimal SOx emissions. Future research, as part of our ongoing work on agricultural wastes, will focus on pyrolysis to produce bio-oil and biochar, as well as co-pyrolysis with polymers. These investigations aim to develop alternative valorisation routes and potentially create synergistic blends that could mitigate the ash-related issues identified in this study.

## Figures and Tables

**Table 1:** Characteristics of the MP sample investigated in this study.

Material and Property	Values (wt%)
Proximate Analysis (wt%)	
Moisture Content	6.0 ± 0.02
Ash	7.55 ± 0.03
Volatile Matter	71.91 ± 0.11
Fixed Carbon	20.53 ± 0.10
Ultimate Analysis (wt%)	
Carbon (C)	44.15 ± 0.16
Hydrogen (H)	6.28 ± 0.03
Oxygen (O)	39.26 ± 0.18
Nitrogen (N)	2.45 ± 0.10
Sulphur (S)	0.33 ± 0.04
Heating Value (MJ kg^−1^)	21.9 ± 0.05
Fiber Fraction (wt%)	
Hemicellulose	12.02 ± 0.04
Cellulose	17.02 ± 0.12
Lignin	10.0 ± 0.05
Metallic Elements (wt% of ash)	
Potassium (K)	34.1 ± 0.10
Calcium (Ca)	16.7 ± 0.08
Silicon (Si)	5.2 ± 0.05
Iron (Fe)	1.3 ± 0.01

**Table 2:** Ash composition of MP by XRF analysis.

Component	Oxide (wt%)	Elemental (wt%)
K_2_O	40.9	K: 34.1
CaO	23.3	Ca: 16.7
SiO_2_	11.1	Si: 5.2
P_2_O_5_	6.4	P: 2.6
SO_3_	6.4	S: 2.6
Al_2_O_3_	5.8	Al: 3.1
FeO/Fe_2_O_3_	1.7–1.9	Fe: 1.19–1.33

**Table 3:** Characteristic temperatures and weight losses (%) for MP combustion.

Heating Rate (K min^−1^)	1st Reaction			2nd Reaction			3rd Reaction		
	T Range, T Peak (K)	Weight Loss %	Process	T Range, T Peak (K)	Weight Loss %	Process	T Range, T Peak (K)	Weight Loss %	Process
20	340–420, 388	6	dehydration	420–538, 495	26	Hemicellulose and cellulose degradation	538–680, 603	35	Cellulose and lignin degradation
40	344–442, 410	5	dehydration	442–560, 518	29	Hemicellulose and cellulose degradation	560–700, 605	35	Cellulose and lignin degradation
60	348–460, 415	3	dehydration	460–580, 520	22	Hemicellulose and cellulose degradation	580–703, 638	47	Cellulose and lignin degradation
80	352–464, 430	4	dehydration	464–590, 550	35	Hemicellulose and cellulose degradation	590–705, 642	33	Cellulose and lignin degradation

**Table 4:** Kinetic parameter values obtained using six model-free methods for MP combustion at different conversion levels.

Conversion	FR		FWO		KAS		STK		K		VY		Average	
	*E_a_* (kJ mol^−1^)	R^2^	*E_a_* (kJ mol^−1^)	R^2^	*E_a_* (kJ mol^−1^)	R^2^	*E_a_* (kJ mol^−1^)	R^2^	*E_a_* (kJ mol^−1^)	R^2^	*E_a_* (kJ mol^−1^)	R^2^	*E_a_* (kJ mol^−1^)	R^2^
0.1	59	0.995	54	0.9997	49	0.9996	49	0.9996	57	0.9997	42	52	59	0.99872
0.2	79	0.998	64	0.9985	59	0.9978	60	0.9978	68	0.9985	47	63	79	0.99812
0.3	138	0.9856	94	0.9937	89	0.9922	90	0.9923	98	0.9937	50	93	138	0.9915
0.4	148	0.9698	143	0.9725	141	0.9687	141	0.9689	151	0.9723	52	129	148	0.97044
0.5	135	0.9871	140	0.9811	137	0.9783	137	0.9784	147	0.9811	103	133	135	0.9812
0.6	290	0.6441	190	0.8468	189	0.832	190	0.8327	200	0.8468	125	197	290	0.8005
Average	142	0.9299	114	0.9654	111	0.9614	111	0.9616	120	0.9654	70	111	111	0.9567

**Table 5:** Pre-exponential factor and thermodynamic parameters of MP combustion.

α	FR					FWO				
	R^2^	A_o_, min^−1^	ΔH kJ mol^−1^	ΔG kJ mol^−1^	ΔS kJ mol^−1^	R^2^	A_o_, min^−1^	ΔH kJ mol^−1^	ΔG kJ mol^−1^	ΔS kJ mol^−1^
0.1	0.9950	3.66 × 10^3^	55.63	131.59	−0.18785	0.9997	1.74 × 10^9^	50.63	82.59	−0.07889
0.2	0.9980	8.84 × 10^5^	74.64	150.29	−0.14402	0.9985	3.84 × 10^10^	59.64	88.67	−0.0553
0.3	0.9856	1.78 × 10^11^	133.64	155.98	−0.04249	0.9937	2.53 × 10^13^	89.64	90.34	−0.00135
0.4	0.9698	2.83 × 10^11^	143.64	163.95	−0.03861	0.9725	3.63 × 10^17^	138.64	97.56	0.07823
0.5	0.9871	1.10 × 10^10^	129.80	171.76	−0.06706	0.9811	6.80 × 10^16^	134.80	95.52	0.06285
0.6	0.6441	1.37 × 10^22^	284.80	182.06	0.16445	0.8468	4.77 × 10^20^	184.80	99.50	0.13649
α	KAS					STK				
	R^2^	A_o_, min^−1^	ΔH kJ mol^−1^	ΔG kJ mol^−1^	ΔS kJ mol^−1^	R^2^	A_o_, min^−1^	ΔH kJ mol^−1^	ΔG kJ mol^−1^	ΔS kJ mol^−1^
0.1	0.9996	1.08 × 10^3^	45.63	125.70	−0.1977	0.9996	1.92 × 10^3^	45.63	123.77	−0.19292
0.2	0.9978	2.10 × 10^4^	54.64	146.61	−0.17519	0.9978	3.69 × 10^4^	55.64	145.15	−0.17051
0.3	0.9922	1.22 × 10^7^	84.64	148.84	−0.12229	0.9923	2.16 × 10^7^	85.64	147.34	−0.11753
0.4	0.9687	1.47 × 10^11^	136.64	159.82	−0.04417	0.9689	2.65 × 10^11^	136.64	157.25	−0.03926
0.5	0.9783	2.51 × 10^10^	131.80	169.49	−0.06029	0.9784	4.54 × 10^10^	131.80	166.41	−0.05536
0.6	0.8320	1.58 × 10^14^	183.80	176.02	0.01245	0.8327	2.86 × 10^14^	184.80	173.94	0.01737
α	K									
	R^2^	A_o_, min^−1^	ΔH kJ mol^−1^	ΔG kJ mol^−1^	ΔS kJ mol^−1^					
0.1	0.9997	5.44 × 10^3^	53.63	128.26	−0.18426					
0.2	0.9985	1.20 × 10^5^	63.64	148.02	−0.16072					
0.3	0.9937	8.02 × 10^7^	93.64	149.61	−0.10662					
0.4	0.9723	1.14 × 10^12^	146.64	160.89	−0.02714					
0.5	0.9811	1.63 × 10^11^	141.80	169.76	−0.04473					
0.6	0.8468	1.14 × 10^15^	194.80	176.75	0.02888					

## Data Availability

All data generated or analysed during this study are included in this published article.

## Supplementary Materials

The following supporting information can be downloaded at: https://www.mdpi.com/article/10.3390/polym17131799/s1. Table S1. Summary of model-free and model-fitting methods used for the kinetic analysis of MP combustion, including their corresponding equations and regression plots (Alhulaybi and Dubdub, 2024) [18]. Table S2. Fifteen of solid–state reaction mechanism (Alhulaybi and Dubdub, 2024) [18]. Table S3. Kinetic parameters obtained by the CR method for MP combustion at four heating rates.

## References

[1] Lopez G., Pourjamal Y., Breyer C. (2025). Paving the Way towards a Sustainable Future or Lagging behind? An Ex-Post Analysis of the International Energy Agency’s World Energy Outlook. Renew. Sustain. Energy Rev..

[2] Mousa S., Dubdub I., Alfaiad M.A., Younes M.Y., Ismail M.A. (2025). Characterization and Kinetic Study of Agricultural Biomass Orange Peel Waste Combustion Using TGA Data. Polymers.

[3] Mohammed I., Abakr Y., Kazi F., Yusup S., Alshareef I., Chin S. (2015). Comprehensive Characterization of Napier Grass as a Feedstock for Thermochemical Conversion. Energies.

[4] Barzegar R., Yozgatligil A., Olgun H., Atimtay A.T. (2020). TGA and Kinetic Study of Different Torrefaction Conditions of Wood Biomass under Air and Oxy-Fuel Combustion Atmospheres. J. Energy Inst..

[5] Arenas C.N., Navarro M.V., Martínez J.D. (2019). Pyrolysis Kinetics of Biomass Wastes Using Isoconversional Methods and the Distributed Activation Energy Model. Bioresour. Technol..

[6] Mango Production by Country 2025. https://worldpopulationreview.com/country-rankings/mango-production-by-country.

[7] Da Guarda Souza M.O., Rebouças L.M., De Castro L.M.F. (2020). Characterization and Thermal Decomposition Study of Mango Residue Biomass (*Mangifera indica* L.). J. Therm. Anal. Calorim..

[8] Liu L., Pang Y., Lv D., Wang K., Wang Y. (2021). Thermal and Kinetic Analyzing of Pyrolysis and Combustion of Self-Heating Biomass Particles. Process Saf. Environ. Prot..

[9] Poletto M., Dettenborn J., Pistor V., Zeni M., Zattera A.J. (2010). Materials Produced from Plant Biomass: Part I: Evaluation of Thermal Stability and Pyrolysis of Wood. Mat. Res..

[10] Nath B., Chen G., Bowtell L., Graham E. (2023). Kinetic Mechanism of Wheat Straw Pellets Combustion Process with a Thermogravimetric Analyser. Heliyon.

[11] Okoroigwe E. (2015). Combustion Analysis and Devolatilazation Kinetics of Gmelina, Mango, Neem and Tropical Almond Woods under Oxidative Condition. Int. J. Renew. Energy Res..

[12] Okoroigwe E.C., Enibe S.O., Onyegegbu S.O. (2016). Determination of Oxidation Characteristics and Decomposition Kinetics of Some Nigerian Biomass. J. Energy South. Afr..

[13] El-Sayed S.A., Mostafa M.E. (2020). Thermal Pyrolysis and Kinetic Parameter Determination of Mango Leaves Using Common and New Proposed Parallel Kinetic Models. RSC Adv..

[14] Tahir M.H., Irfan R.M., Cheng X., Ahmad M.S., Jamil M., Shah T.-U.-H., Karim A., Ashraf R., Haroon M. (2021). Mango Peel as Source of Bioenergy, Bio-Based Chemicals via Pyrolysis, Thermodynamics and Evolved Gas Analyses. J. Anal. Appl. Pyrolysis.

[15] Yousef S., Eimontas J., Striūgas N., Abdelnaby M.A. (2021). Pyrolysis and Gasification Kinetic Behavior of Mango Seed Shells Using TG-FTIR-GC–MS System under N_2_ and CO_2_ Atmospheres. Renew. Energy.

[16] Alves J.L.F., Da Silva J.C.G., Mumbach G.D., Alves R.F., Di Domenico M., Marangoni C. (2023). Physicochemical Properties, Pyrolysis Kinetics, Thermodynamic Parameters of Activation, and Evolved Volatiles of Mango Seed Waste as a Bioenergy Feedstock: A Potential Exploration. Thermochim. Acta.

[17] D05 Committee ASTM International (2004). Test Method for Ash in the Analysis Sample of Coal and Coke from Coal.

[18] Alhulaybi Z.A., Dubdub I. (2024). Kinetics Study of PVA Polymer by Model-Free and Model-Fitting Methods Using TGA. Polymers.

[19] Ou C., Chen S., Liu Y., Shao J., Li S., Fu T., Fan W., Zheng H., Lu Q., Bi X. (2016). Study on the Thermal Degradation Kinetics and Pyrolysis Characteristics of Chitosan-Zn Complex. J. Anal. Appl. Pyrolysis.

[20] Coats A.W., Redfern J.P. (1964). Kinetic Parameters from Thermogravimetric Data. Nature.

[21] Galwey A.K. (2003). Eradicating Erroneous Arrhenius Arithmetic. Thermochim. Acta.

[22] Tariq R., Mohd Zaifullizan Y., Salema A.A., Abdulatif A., Ken L.S. (2022). Co-Pyrolysis and Co-Combustion of Orange Peel and Biomass Blends: Kinetics, Thermodynamic, and ANN Application. Renew. Energy.

[23] Yao Z., Romano P., Fan W., Gautam S., Tippayawong N., Jaroenkhasemmeesuk C., Liu J., Wang X., Qi W. (2024). Probing Pyrolysis Conversion of Separator from Spent Lithium-Ion Batteries: Thermal Behavior, Kinetics, Evolved Gas Analysis and Aspen plus Modeling. Case Stud. Therm. Eng..

[24] Tong W., Cai Z., Liu Q., Ren S., Kong M. (2020). Effect of Pyrolysis Temperature on Bamboo Char Combustion: Reactivity, Kinetics and Thermodynamics. Energy.

[25] Huang L., Liu J., He Y., Sun S., Chen J., Sun J., Chang K., Kuo J., Ning X. (2016). Thermodynamics and Kinetics Parameters of Co-Combustion between Sewage Sludge and Water Hyacinth in CO2/O2 Atmosphere as Biomass to Solid Biofuel. Bioresour. Technol..

[26] Liu H., Wang C., Zhang J., Zhao W., Fan M. (2020). Pyrolysis Kinetics and Thermodynamics of Typical Plastic Waste. Energy Fuels.

[27] Kalidasan B., Pandey A.K., Aljafari B., Chinnasamy S., Kareri T., Rahman S. (2023). Thermo-Kinetic Behaviour of Green Synthesized Nanomaterial Enhanced Organic Phase Change Material: Model Fitting Approach. J. Environ. Manag..

