# Supplementary material for: Characterization, Combustion Behaviour, and Kinetic and Thermodynamic Modelling of Mango Peel as a Potential Biomass Feedstock
Source: Polymers (Basel). 2025 Jun 27;17(13):1799. doi: 10.3390/polym17131799 (PMC12251654; doi:10.3390/polym17131799)
Supplement: Supplementary file 1 [file polymers-17-01799-s001.zip › polymers-3697891-supplementary.pdf]

polymers

MDPI

Supplementary Materials

Characterization, Combustion Behaviour, and Kinetic and Thermodynamic Modelling of Mango Peel as a Potential Biomass Feedstock

Mohamed Anwar Ismail [1], Ibrahim Dubdub [2,*], Suleiman Mousa [2], Zaid Abdulhamid Alhulaybi Albin Zaid [2,*] and Majdi Ameen Alfaiad [2]

[1] Mechanical Engineering Department, King Faisal University, Al-Ahsa 31982, Saudi Arabia; maismail@kfu.edu.sa
[2] Chemical Engineering Department, King Faisal University, Al-Ahsa 31982, Saudi Arabia; saamousa@kfu.edu.sa (S.M.); malfaiad@kfu.edu.sa (M.A.A.)
* Correspondence: idubdub@kfu.edu.sa (I.D.); zalhulaybi@kfu.edu.sa (Z.A.A.A.Z.); Tel.: +966-13-589-6989 (I.D.)

Table S1. Summary of model-free and model-fitting methods used for the kinetic analysis of MP combustion, including their corresponding equations and regression plots (Alhulaybi and Dubdub, 2024).

model-free methods		
**Method**	**Formula**	**Plot**
FR	$ln\left(\beta\frac{d\alpha}{dT}\right) = ln[A_o f(\alpha)] - \frac{E_a}{RT}$ (4)	$ln\left(\beta\frac{d\alpha}{dT}\right) vs. \frac{1}{T}$
FWO	$ln(\beta) = ln\frac{A_o E_a}{Rg(\alpha)} - 5.331 - 1.052\frac{E_a}{RT}$ (5)	$ln(\beta)\ vs.\ \frac{1}{T}$
KAS	$ln\left(\frac{\beta}{T^2}\right) = ln\frac{A_o R}{E_a\, g(\alpha)} - \frac{E_a}{RT}$ (6)	$ln\left(\frac{\beta}{T^2}\right) vs. \frac{1}{T}$
STK	$ln\frac{\beta}{T^{1.92}} = ln\left(\frac{A_0 E_a}{Rg(\alpha)}\right) - 1.0008\frac{E_a}{RT}$ (7)	$ln\frac{\beta}{T^{1.92}} vs. \frac{1}{T}$
K	$\ln\left(\frac{\beta}{T_m^2}\right) = ln\left(\frac{A_0 R}{E_a}\right) - \frac{E_a}{RT}$ (8)	$\ln\left(\frac{\beta}{T_m^2}\right) vs. \frac{1}{T}$
VY	$\Phi(E_\alpha) = \sum_{i=1}^{n}\sum_{j\neq i}^{n}\frac{J[E_\alpha, T_i(t_\alpha)]}{J[E_\alpha, T_j(t_\alpha)]} = 0$ (9)	minimizing the function $\Phi(E_\alpha)$
model-fitting methods		
**Method**	**Formula**	**Plot**
CR	$ln\left[\frac{g(\alpha)}{T^2}\right] = ln\left[\frac{A_o R}{\beta E_a}\right] - \frac{E}{RT}$ (10)	$ln\left[\frac{g(\alpha)}{T^2}\right] vs. \frac{1}{T}$

Table S2. Fifteen of solid–state reaction mechanism (Alhulaybi and Dubdub, 2024).

Reaction mechanism	Code	$f(\alpha)$	$g(\alpha)$
Reaction order models-1st order	F1	1-α	$-\ln(1-\alpha)$
Reaction order models-2ndorder	F2	$(1-\alpha)^2$	$(1-\alpha)^{-1}-1$
Reaction order models-3rd order	F3	$(1-\alpha)^3$	$[(1-\alpha)^{-1}-1]/2$
Diffusion model-1 dimension	D1	$1/2\alpha^{-1}$	α^2
Diffusion model-2-dimension	D2	$[-ln\,(1-\alpha)]^{-1}$	$(1-\alpha)\ln(1-\alpha)+\alpha$
Diffusion model-3-dimension	D3	$3/2[1-(1-\alpha)^{1/3}]^{-1}$	$[1-(1-\alpha)^{1/3}]^2$
Diffusion model-4-dimension	D4	1.5*((1-α)^(-1/3)-1)	1-(2/3)*α-(1-α)^(2/3)
Nucleation models-2 dimension	A2	$2(1-\alpha)[-\ln(1-\alpha)]^{1/2}$	$[-\ln(1-\alpha)]^{1/2}$
Nucleation models-3-dimension	A3	$3(1-\alpha)[-\ln(1-\alpha)]^{1/3}$	$[-\ln(1-\alpha)]^{1/3}$
Nucleation models-4-dimension	A4	$4(1-\alpha)[-\ln(1-\alpha)]^{1/4}$	$[-\ln(1-\alpha)]^{1/4}$
Geometrical contraction models-1-dimension	R1	1	α
Geometrical contraction models - sphere	R2	$2(1-\alpha)^{1/2}$	1-$(1-\alpha)^{1/2}$
Geometrical contraction models - cylinder	R3	$3(1-\alpha)^{1/3}$	1-$(1-\alpha)^{1/3}$
Nucleation models-2-Power law	P2	$2\alpha^{1/2}$	$\alpha^{1/2}$
Nucleation models-3-Power law	P3	$3\alpha^{2/3}$	$\alpha^{1/3}$
Nucleation models-4-Power law	P4	$4\alpha^{3/4}$	$\alpha^{1/4}$

Table S3. Kinetic parameters obtained by the CR method for MP combustion at four heating rates.

**Reaction mechanism 1 step reaction**	**Code**	**20**			**40**			**60**		
		**E_a (kJ/mol)**	**Ln(A_0)**	**R^2**	**E_a (kJ/mol)**	**Ln(A_0)**	**R^2**	**E_a (kJ/mol)**	**Ln(A_0)**	**R^2**
Reaction order models-First order	F1	29	17.34	0.996	33	17.74	0.9967	31	3.43	0.9986
Reaction order models-Second order	F2	30	17.18	0.9962	34	17.63	0.9968	32	3.47	0.9987
Reaction order models-Third order	F3	30	16.98	0.9965	34	17.5	0.997	32	3.47	0.9988
Diffusion models-One dimension	D1	63	13.06	0.9967	73	14.96	0.9972	69	4.23	0.9989
Diffusion models-Two dimension	D2	64	12.51	0.9967	73	14.35	0.9972	69	4.23	0.9989
**Diffusion models-Three dimension**	**D3**	**64**	**12.76**	**0.9968**	**73**	**12.93**	**0.9973**	**70**	**4.25**	**0.9989**
Diffusion models-Four dimension	D4	64	12.84	0.9967	73	12.88	0.9973	69	4.23	0.9989
Nucleation models-Two dimension	A2	11	20.22	0.9931	13	20.9	0.9945	12	2.48	0.9976
Nucleation models-Three-dimension	A3	5	20.71	0.9861	7	21.66	0.9897	6	1.79	0.995
Nucleation models Fourth dimension	A4	2	20.44	0.9618	3	21.5	0.9763	3	1.10	0.9855
Geometrical contraction models-One dimension phase boundary	R1	28	17.49	0.9958	22	17.46	0.9965	31	3.43	0.9986
Geometrical contraction models -Contracting sphere	R2	29	18.13	0.9959	33	18.49	0.9966	31	3.43	0.9986
Geometrical contraction models- Contracting cylinder	R3	29	18.5	0.9959	33	18.88	0.9966	31	3.43	0.9986
Nucleation models-Power law	P2	11	20.31	0.9926	13	20.98	0.9942	12	2.48	0.9974
Nucleation models-Power law	P3	5	20.78	0.9848	7	21.7	0.9891	6	1.79	0.9946
Nucleation models-Power law	P4	2	20.49	0.9565	3	21.53	0.9743	3	1.10	0.9837

**Reaction mechanism1 step reaction**	**Code**	**80**		
		**E_a (kJ/mol)**	**Ln(A_0)**	**R^2**
Reaction order models-First order	F1	28	20.34	0.9971
Reaction order models-Second order	F2	29	20.22	0.9973
Reaction order models-Third order	F3	29	20.07	0.9974
Diffusion models-One dimension	D1	63	15.15	0.9977
Diffusion models-Two dimension	D2	63	15.74	0.9977
**Diffusion models-Three dimension**	**D3**	**64**	**17.16**	**0.9978**

Diffusion models-Four dimension	D4	63	17.21	0.9978
Nucleation models-Two dimension	A2	11	22.55	0.9945
Nucleation models-Three-dimension	A3	5	22.81	0.9868
Nucleation models Fourth dimension	A4	2	22.42	0.9433
Geometrical contraction models-One dimension phase boundary	R1	28	20.49	0.997
Geometrical contraction models -Contracting sphere	R2	28	21.1	0.9971
Geometrical contraction models- Contracting cylinder	R3	28	21.49	0.9971
Nucleation models-Power law	P2	10	22.53	0.9942
Nucleation models-Power law	P3	5	22.86	0.9856
Nucleation models-Power law	P4	2	22.46	0.9339

**Reaction mechanism 2 step reaction**	**Code**	**20**			**40**			**60**		
		**E_a (kJ/mol)**	**Ln(A_0)**	**R^2**	**E_a (kJ/mol)**	**Ln(A_0)**	**R^2**	**E_a (kJ/mol)**	**Ln(A_0)**	**R^2**
Reaction order models-First order	F1	43	15.35	0.9928	49	15.07	0.9983	45	16.62	0.9955
Reaction order models-Second order	F2	49	13.83	0.9945	54	13.91	0.9981	52	14.89	0.9967
Reaction order models-Third order	F3	56	12.19	0.9957	59	12.67	0.9976	61	13.01	0.9975
Diffusion models-One dimension	D1	82	16.01	0.9925	96	19.73	0.9985	84	16.61	0.9952
Diffusion models-Two dimension	D2	86	16.34	0.9931	99	19.84	0.9985	89	17.07	0.9956
**Diffusion models-Three dimension**	**D3**	**90**	**15.9**	**0.9936**	**103**	**19.17**	**0.9985**	**93**	**16.76**	**0.996**
Diffusion models-Four dimension	D4	87	15.19	0.9932	100	18.61	0.9985	90	15.96	0.9957
Nucleation models-Two dimension	A2	17	19.75	0.9885	20	20.05	0.9975	18	21.03	0.9928
Nucleation models-Three-dimension	A3	9	20.9	0.9794	11	21.41	0.9961	9	22.11	0.9869
Nucleation models Fourth dimension	A4	4	20.97	0.9543	6	21.78	0.9928	4	22.19	0.9699
Geometrical contraction models-One dimension phase boundary	R1	37	16.72	0.9906	44	16.14	0.9981	38	18.17	0.9939
Geometrical contraction models -Contracting sphere	R2	40	16.75	0.9917	46	16.3	0.9983	41	18.10	0.9948
Geometrical contraction models- Contracting cylinder	R3	41	16.92	0.9921	47	16.52	0.9983	42	18.24	0.995
Nucleation models-Power law	P2	1	17.68	0.9879	18	20.54	0.9971	14	21.64	0.9892
Nucleation models-Power law	P3	7	21.15	0.9666	10	21.71	0.995	7	22.43	0.9768
Nucleation models-Power law	P4	3	21.07	0.9025	5	21.9	0.9893	3	22.33	0.9229

**Reaction mechanism 2 step reaction**	**Code**	**80**		
		**E_a (kJ/mol)**	**Ln(A_0)**	**R^2**
Reaction order models-First order	F1	59	14.23	0.9983
Reaction order models-Second order	F2	67	14.22	0.9991
Reaction order models-Third order	F3	75	16.28	0.9995
Diffusion models-One dimension	D1	113	23.13	0.9976
Diffusion models-Two dimension	D2	118	23.57	0.998
**Diffusion models-Three dimension**	**D3**	**122**	**23.24**	**0.9983**
Diffusion models-Four dimension	D4	119	22.46	0.9981
Nucleation models-Two dimension	A2	25	21.18	0.9976
Nucleation models-Three-dimension	A3	14	21.86	0.9962
Nucleation models Fourth dimension	A4	8	22.44	0.9936
Geometrical contraction models-One dimension phase boundary	R1	52	15.8	0.9972
Geometrical contraction models -Contracting sphere	R2	56	15.74	0.9978
Geometrical contraction models- Contracting cylinder	R3	57	15.88	0.998
Nucleation models-Power law	P2	22	20.9	0.9957
Nucleation models-Power law	P3	11	22.19	0.9929
Nucleation models-Power law	P4	6	22.58	0.9864

**Reaction mechanism 3 step reaction**	**Code**	**20**			**40**			**60**		
		**E_a (kJ/mol)**	**Ln(A_0)**	**R^2**	**E_a (kJ/mol)**	**Ln(A_0)**	**R^2**	**E_a (kJ/mol)**	**Ln(A_0)**	**R^2**
Reaction order models-First order	F1	20	19.86	0.9974	24	20.18	0.9971	29	19.83	0.998
Reaction order models-Second order	F2	31	17.66	0.9958	37	17.72	0.996	49	16.14	0.9992
Reaction order models-Third order	F3	45	14.91	0.9945	52	14.65	0.9951	74	14.97	0.9996
Diffusion models-One dimension	D1	32	18.93	0.9995	38	18.99	0.9991	39	19.18	0.9964
Diffusion models-Two dimension	D2	37	18.5	0.9991	44	18.45	0.9987	48	17.82	0.9973
**Diffusion models-Three dimension**	**D3**	**44**	**18.7**	**0.9986**	**51**	**18.49**	**0.9983**	**58**	**17.72**	**0.9981**
Diffusion models-Four dimension	D4	39	19.56	0.999	46	19.46	0.9986	51	19.09	0.9976

Nucleation models-Two dimension	A2	5	21.32	0.9914	7	22.1	0.9922	9	22.33	0.9947
Nucleation models-Three-dimension	A3	NA	NA	NA	1	21.21	0.9059	3	22.46	0.9697
Nucleation models Fourth dimension	A4	NA	NA	NA	NA	NA	NA	NA	NA	NA
Geometrical contraction models-One dimension phase boundary	R1	11	21.41	0.9991	14	21.97	0.9984	14	22.30	0.993
Geometrical contraction models -Contracting sphere	R2	15	21.4	0.9982	19	21.87	0.9978	21	21.92	0.9964
Geometrical contraction models- Contracting cylinder	R3	17	21.57	0.9979	20	21.93	0.9976	24	21.91	0.9971
Nucleation models-Power law	P2	1	20.78	0.9626	2	22.02	0.9811	2	22.43	0.8959
Nucleation models-Power law	P3	NA	NA	NA	NA	NA	NA	NA	NA	NA
Nucleation models-Power law	P4	NA	NA	NA	NA	NA	NA	NA	NA	NA

Reaction mechanism1 step reaction	Code	80		
		E_a (kJ/mol)	Ln(A_0)	R^2
Reaction order models-First order	F1	29	20.28	0.9977
Reaction order models-Second order	F2	46	17.17	0.9965
Reaction order models-Third order	F3	66	13.48	0.9956
Diffusion models-One dimension	D1	42	19.11	0.9993
Diffusion models-Two dimension	D2	50	18.34	0.9991
**Diffusion models-Three dimension**	**D3**	**59**	**18.06**	**0.9987**
Diffusion models-Four dimension	D4	53	19.26	0.9989
Nucleation models-Two dimension	A2	9	22.69	0.9947
Nucleation models-Three-dimension	A3	3	22.79	0.9732
Nucleation models Fourth dimension	A4	NA	NA	NA
Geometrical contraction models-One dimension phase boundary	R1	16	22.51	0.9989
Geometrical contraction models -Contracting sphere	R2	22	22.2	0.9983
Geometrical contraction models- Contracting cylinder	R3	24	22.21	0.9981
Nucleation models-Power law	P2	3	23	0.9921
Nucleation models-Power law	P3	NA	NA	NA
Nucleation models-Power law	P4	NA	NA	NA

*NA: Not available.
